# Building a Physician/Advanced Practice Provider Partnership for Inpatient Dialysis Care

**DOI:** 10.34067/KID.0000000633

**Published:** 2024-10-28

**Authors:** Rita L. McGill, Tammy Poma, Arlene B. Chapman

**Affiliations:** Section of Nephrology, Department of Medicine, University of Chicago, Chicago, Illinois

**Keywords:** chronic hemodialysis, hemodialysis, hospitalization, outcomes, supportive care

More than 550,000 patients in the United States receive hemodialysis, many of whom require inpatient care because of frailty and cardiovascular disease.^1,2^ Increased inpatient volumes of dialysis-dependent patients strain health systems and providers because the nephrology workforce has not expanded.^3,4^ At the University of Chicago Medical Center (UCMC), we implemented a collaborative care model between attending nephrologists, nephrology fellows, and advanced practice providers (APPs), aiming to improve service and patient experience while enhancing fellowship education and reducing provider burnout.

UCMC provides 600–800 inpatient hemodialyses per month to patients in two buildings 200 yards apart, the Center for Care and Discovery (CCD) and Mitchell Hospital, serving 400–500 patients undergoing hemodialysis at UCMC-affiliated outpatient facilities and hundreds more at other nearby facilities. On a typical day, the inpatient census is 30–40 patients with 12–18 hemodialysis treatments, 5–8 new consultations during daylight hours, and 1–4 additional cases arising in the emergency department overnight. Our preexisting model staffed the service with a single nephrologist supervising a single nephrology fellow. Coverage was provided by a division of labor strategy, with separate workflows for the attending nephrologist and the trainee. Fellows concentrated on producing initial consult notes, and attendings focused on providing dialysis treatment oversight. Documentation on nondialysis days was inconsistent, with loss of revenue and suboptimal communication to our colleagues. This arrangement was unsatisfactory to all stakeholders. Separate workflows compromised the proximity of the attending and the fellow, reducing the frequency and quality of teaching. The rotation was consistently reported to the training program as onerous, with a suboptimal ratio of service to education. The rotation was similarly unpopular with attending physicians, who found the assignment so arduous that service was largely limited to no more than 7 days at a time. Referring teams were often uncertain of the dialysis plans because of the dearth of notes on nondialysis days, which manifested as constant paging of the dialysis fellow and frequent telephone calls to the dialysis reception desk. The dialysis staff were frustrated by the endless telephone inquiries and concerned when providers rounding at Mitchell left them without visible support in the dialysis suite.

Opportunities for Advanced Practice Provider (APPs) in nephrology increased in the wake of 2004 Centers for Medicare & Medicaid Services regulations designed to increase the frequency of face-to-face visits in outpatient dialysis facilities.^5^ Nephrology APPs typically practice in the outpatient dialysis setting or the nephrology clinic and less commonly provide hospital care.^6–8^ UCMC had already incorporated APPs successfully in other settings.^9^ In Illinois, advanced practice nurses (APNs) are required to begin practice with a collaborating physician, but Section 225 Illinois Compiled Statutes 65, also known as the Nurse Practice Act, allows an Illinois-licensed advanced practice registered nurse (APN) certified as a nurse practitioner to practice without a written collaborative agreement after completing 250 hours of continuing education/training and 4000 hours of clinical experience after national certification.^10^ We initiated our program with the four APNs from our outpatient dialysis facilities. Each UCMC dialysis program is staffed by an medical director/APN pair who share responsibilities for patient care and documentation. Each of these APNs has a Master's degree in nursing and two or more years' experience as a dialysis nurse and had provided direct patient care in a UCMC dialysis facility for at least 3 years, covering the hemodialysis competencies necessary in the inpatient setting. We felt that onboarding an APP in the outpatient or inpatient setting would be prohibitive for anyone not intimately experienced with dialysis care because of hemodialysis-specific issues, such as intradialytic hypotension and vascular access. Each APN was trained by their respective facility medical director, with whom they had a formal collaborative agreement. The APNs had experience using the UCMC electronic health record (Epic) for prescribing and scheduling vascular access procedures and access to the DaVita electronic medical record (OneView). The fellows and APNs already knew one another due to the fellowship's outpatient dialysis rotations. We were fortunate that the APNs found in-hospital rounds to be a change of pace that brought them face to face with people with whom they had extensively interacted with by telephone.

We initiated our collaborative model in June 2021, providing APN coverage from Wednesday through Saturday. We developed extensive written training materials, to define five tasks of sequential complexity, allowing responsibilities to evolve with comfort levels and expertise (Table 1). The first task was to supervise inpatient hemodialysis within the dialysis suite and provide procedural documentation for dialysis treatments. The APNs achieved this immediately, and the acute care dialysis staff appreciated the high-quality coverage. Relief from dialysis notes provided the nephrology attending with time to teach the fellow. For the first month or two, bedside rounds in the Mitchell building with the APN were performed in the morning, followed by rounds in CCD with the fellow. Fellows initially wrote all new consultations and dialysis orders, but within 2–3 months, the APNs were comfortable expanding their role to writing dialysis orders and off-day progress notes, conveying care plans to referring services. This expansion of their role provided valuable relief to our weekend on-call teams and the inpatient dialysis reception desk.

**Table 1:** Inpatient dialysis service tasks

Work Assignment
1. Supervision of acute hemodialysis treatments
2. Evaluation and management
3. Hemodialysis orders
4. Initial consultation notes
5. Service sign-outs

Provision of initial consult notes was an individualized process because the training and experience of the APNs was variable. We allowed ourselves to be guided by APN comfort levels. Two of our APNs were performing independent initial consults within the first year. A more standardized Epic template for documentation of dialysis consults was developed to guide both fellows and APNs to compose more complete and helpful consultations, with cues for thoughtful kidney-specific care with a minimum of pasted information. This template enabled the remaining two APNs to become adept over the next 3 months. Training materials evolved in response to changing responsibilities and focused on both dialysis-specific tasks and systems approaches to interacting with the other hospital stakeholders. These training materials have been valuable for new fellows (Table 2).

**Table 2:** Reference and training materials

Contents
**General logistics**
Building maps
Locations of important services
Accessing schedules
Census lists
Email, paging, and secure text communication
**Documentation and billing**
Available Epic note templates/customization
Hemodialysis procedures
Dialysis consults/progress notes
*Present illness, interval history, and assessment/Plans*
*Sign-outs and transitions to/from outpatient care*
*Documentation integrity in the age of electronic records*
**Results**
Laboratory results
X-ray films and reports
Customized data entry into progress notes
**Vascular access (how to)**
Interventional radiology
Vascular laboratory
Vascular surgery
Anticoagulation
Contrast allergies
Dialysis center phone/fax numbers

The transition to an inpatient acute dialysis setting required considerable adaptation, even for APNs with years of experience in the outpatient setting. The APNs were new to the academic hospital setting and had to navigate a brand-new team dynamic. Problems that would have been managed directly in an outpatient facility now relied on communication to a primary medical service that had ownership of the ordering process, with a challenging obligation to follow up and ensure that care was coordinated as intended. Working in the outpatient dialysis setting gave the APNs the capacity to know their own patients' medical histories and the particulars of their day-to-day treatments intimately, such as ultrafiltration tolerance and the nuances of each particular fistula. In the hospital, the patient population rotated rapidly and many patients were completely unfamiliar, giving our APNs new understanding into the motivations of our inpatient dialysis teams.

Rounding in the hospital reduced the work-hour flexibility of the APNs, and Saturday coverage was a new expectation. We left it to each medical director/APN dyad to determine when the APN could take a day off from their outpatient facility, to compensate for the extra Saturday work. We did not attempt to cover holidays and had APNs elect one of their own cohort to manage their schedule. An attending with concerns was allowed to opt-out of APN coverage, a phenomenon that quickly ceased. The APNs practiced under their existing collaborative agreements.

After 1 year, we established a full-time inpatient APN position with a collaborating physician, increasing service coverage to 6 days per week. With roughly equal censuses in the two buildings, it was logical to divide service into APN-Mitchell and Fellow-CCD. These borders were flexible, with fellows and APNs ready to help each other. Inpatient peritoneal dialysis cases were reserved for the fellows, for training purposes. On Saturdays, the APN assumed responsibility for patients on hemodialysis in both buildings. Although individual nephrologists have their own preferences, the service typically meets for 45–60 minutes each morning to discuss all patients. The APN rounds independently and writes all dialysis notes/orders, progress notes, and new consultations for patients admitted to the Mitchell building. The fellow and attending perform mutual bedside rounds on all patients in the CCD. The attending writes all dialysis notes for CCD, reviews and countersigns the fellow's progress notes and consults, and reviews APN documentation. On a typical day, the APN delivers two to four new consults and rounds on 10–15 patients, half of whom receive progress notes and half of whom require supervision of dialysis treatments. The fellow produces six to eight new consults and rounds on 10–15 follow-up patients. The inpatient APN position turned over once, and an outpatient dialysis APN retired and was replaced by a physician assistant with extensive experience in hemodialysis, redefining our project as an APP service. Our experienced cadre of APNs was ready to take an active role in training the new APPs. The UCMC credentialing processes provided new hires with a 3- to 5-month period of shadowing, from which new recruits emerged ready and eager for active rounding. We use a 6-week rotation, with four facility APPs and one inpatient APN. The four facility-based APPs cover 2–3 days every 6 weeks; the inpatient APN covers Monday through Thursday during 4 of the 6 weeks and Tuesday through Saturday during the other two. The APP service does not cover Sundays, when outpatient dialysis facilities are closed and inpatient volume is low. The inpatient APN covers one holiday per year, similar to fellows. Clinical volumes are lower during holidays, and APP support has been more useful during the postholiday re-entry period. The rotation system taught everyone that high-quality transitions required diligent sign-out, which is taught explicitly as part of the orientation to the inpatient dialysis service.

Three years later, we can appreciate that the combined service provides better documentation with more effective communication of our treatment plans. Complaints from referring services have virtually ceased. The APP rounds and bills independently but can request attending support, a model which had already been developed for other UCMC service lines. The fellow and the APP both have access to the attending nephrologist, who has more time to teach. Rounding on the service is easier for both fellows and attendings, and for most faculty, the relative value units absorbed by APPs has been offset by the increased frequency of progress notes. Attendings have the option of countersigning APN visits and billing for them, as long as they can attest to having been present at the bedside to perform the key parts of the visit, but this option has been only rarely invoked.

We find that the APPs are valued members of our team. Inpatients are often delighted to encounter their APP during hospitalizations. The APPs anticipate issues that complicate transfer from hospital to an outpatient setting; their insights often streamline discharge planning. The fellows and the APPs bring different assets to the team, and the APPs have been valuable guides for entering fellows. Having the fellows and APPs work side by side has also facilitated more meaningful experiences for the fellows rotating in the outpatient dialysis facilities, so the academic mission has been enhanced as much as the clinical mission. The personal approach of the APPs, who are accustomed to spending more time with their patients, enriches patient experience and sets a model of communication for our physicians in training. Our fellows capitalize on this, now that the workload does not dictate such a frantic pace.

In summary, the expertise that APPs bring to outpatient dialysis translates well into high-quality hospital care of patients on dialysis, with advantages to patients, providers, and trainees.
